# CXCL10 Can Inhibit Endothelial Cell Proliferation Independently of CXCR3

**DOI:** 10.1371/journal.pone.0012700

**Published:** 2010-09-13

**Authors:** Gabriele S. V. Campanella, Richard A. Colvin, Andrew D. Luster

**Affiliations:** Division of Rheumatology, Allergy and Immunology, Center for Immunology and Inflammatory Diseases, Massachusetts General Hospital, Harvard Medical School, Charlestown, Massachusetts, United States of America; Virginia Commonwealth University, United States of America

## Abstract

CXCL10 (or Interferon-inducible protein of 10 kDa, IP-10) is an interferon-inducible chemokine with potent chemotactic activity on activated effector T cells and other leukocytes expressing its high affinity G protein-coupled receptor CXCR3. CXCL10 is also active on other cell types, including endothelial cells and fibroblasts. The mechanisms through which CXCL10 mediates its effects on non-leukocytes is not fully understood. In this study, we focus on the anti-proliferative effect of CXCL10 on endothelial cells, and demonstrate that CXCL10 can inhibit endothelial cell proliferation *in vitro* independently of CXCR3. Four main findings support this conclusion. First, primary mouse endothelial cells isolated from CXCR3-deficient mice were inhibited by CXCL10 as efficiently as wildtype endothelial cells. We also note that the proposed alternative splice form CXCR3-B, which is thought to mediate CXCL10's angiostatic activity, does not exist in mice based on published mouse CXCR3 genomic sequences as an in-frame stop codon would terminate the proposed CXCR3-B splice variant in mice. Second, we demonstrate that human umbilical vein endothelial cells and human lung microvascular endothelial cells that were inhibited by CXL10 did not express CXCR3 by FACS analysis. Third, two different neutralizing CXCR3 antibodies did not inhibit the anti-proliferative effect of CXCL10. Finally, fourth, utilizing a panel of CXCL10 mutants, we show that the ability to inhibit endothelial cell proliferation correlates with CXCL10's glycosaminoglycan binding affinity and not with its CXCR3 binding and signaling. Thus, using a very defined system, we show that CXCL10 can inhibit endothelial cell proliferation through a CXCR3-independent mechanism.

## Introduction

Chemokines are a superfamily of chemotactic cytokines, which play important roles in the generation and delivery of immune and inflammatory responses [1]–[3]. They orchestrate the movement of leukocytes and other cells by activating specific seven-transmembrane spanning G protein-coupled receptors expressed on responsive cells. CXCL10, or IP-10 (Interferon-induced protein of 10 kDa), one of the first chemokines identified [4], [5], directs the trafficking of activated effector CD4^+^ and CD8^+^ T lymphocytes and other effector lymphocytes, such as NK and NKT cells [6]–[9]. It does so by binding its high affinity receptor CXCR3 [10], [11], which it shares with two other ligands, monokine-induced by γ-interferon (Mig/CXCL9) and interferon-inducible T cell-α chemoattractant (I-TAC/CXCL11). CXCL10 expression is strongly up-regulated in many human inflammatory diseases, including viral, bacterial and parasitic infections, skin diseases, atherosclerosis, allograft rejection, and others [1].

In addition to its role in the activation and recruitment of effector T cells and other leukocytes, CXCL10 acts on other cell types, in particular on endothelial cells. Indeed, among the first described functions of CXCL10 were its anti-proliferative effect on endothelial cells *in vitro* [12], and its angiostatic [13]–[15] and anti-tumor effect *in vivo* [6], [16]. *In vivo* CXCL10 inhibits neovascularization in tumors as well as wound healing [17]–[21]. The mechanisms by which CXCL10 exerts its effects on endothelial cells have remained elusive and in some instances controversial.

The identification of an alternative splice variant of CXCR3, termed CXCR3-B, specifically in human endothelial cells, was suggested as a possible explanation for CXCL10's angiostatic effects [22]. Translation of the putative human CXCR3-B splice variant results in an extracellular N-terminus that is 48 amino acids longer than the originally described CXCR3 receptor (referred to as CXCR3-A), with the remaining 3′ sequence identical to CXCR3-A. The traditional CXCR3 ligands, CXCL10, 9 and 11, were shown to bind to CXCR3-B. In addition, CXCL4 (Platelet Factor 4, PF4), was also shown to weakly bind CXCR3-B. CXCR3-B has been described to mediate the angiostatic effect of its ligands, being the preferential CXCR3 receptor reported to be expressed on endothelial cells. Strikingly, overexpression of CXCR3-B in an endothelial cell line resulted in CXCL10 inhibiting proliferation, whereas overexpression of CXCR3-A in the same cell line resulted in CXCL10 augmenting proliferation [22].

Although the existence of an alternative splice variant CXCR3 provides a possible explanation for the different functions of CXCL10, it is unclear how a difference in only the N-terminal extracellular domain of CXCR3-A results in intracellular signaling that was purported to oppose CXCR3-A signaling. In addition, it has been unclear whether CXCR3-B exists in rodents, although CXCL10's anti-proliferative effects on endothelial cells have been described in mice. We therefore utilized a very defined *in vitro* system to address whether the anti-proliferative effect of CXCL10 on endothelial cells is mediated through CXCR3. We demonstrate that CXCL10 was capable of inhibiting the proliferation of murine endothelial cells that were deficient in CXCR3. Furthermore, we show that the alternative CXCR3-B variant does not exist in mice, as an in-frame stop codon before the conserved sequence would terminate an analogous CXCR3-B splice variant in mice. Similarly, our experiments with human endothelial cells also demonstrate that CXCL10 can inhibit endothelial cell proliferation independently of CXCR3.

## Results

### CXCL10 inhibits proliferation of endothelial cells isolated from different tissue

**Figure 1:**
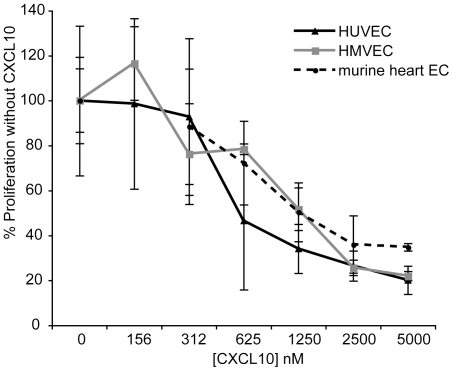
CXCL10 inhibits proliferation of endothelial cells isolated from different organs. Human umbilical cord vein endothelial cells (HUVEC), human lung microvascular endothelial cells (HMVEC-L), and murine heart endothelial cells isolated from C57Bl/6 mice were plated in 96-well plates with bFGF. 24 hr after seeding, new media with CXCL10 was added and replaced every 48 hr for a total of 3–4 days. ^3^H-thymidine incorporation was assessed for the final 18 hr of incubation. Representative data of at least three experiments are shown, each performed in triplicate, data are mean ± SD.

We tested CXCL10's effect on the proliferation of three primary endothelial cell types, human umbilical cord endothelial cells (HUVEC), human lung microvascular endothelial cells (HMVEC-L), and murine heart endothelial cells isolated from C57Bl/6 mice. As has been described [12], at relatively high concentrations, CXCL10 inhibited bFGF-induced proliferation for all three endothelial cell types, with concentrations of 0.5–1.0 µM CXCL10 inhibiting 50% of proliferation, whereas 5.0 µM inhibited 80% of proliferation (Fig. 1). The IC_50_ value for CXCL10's antiproliferative effect therefore is between 0.5–1.0 µM, similar to Lasagni et al. who reported peak inhibition of HMVEC proliferation at 100 nM–1 µM CXCL10 [22], and similar to the IC_50_ value of ∼150 nM that we previously reported [12]. These results demonstrate that at relatively high concentrations, CXCL10 can inhibit multiple types of endothelial cells.

### CXCL10 anti-proliferative effect on murine endothelial cells is CXCR3 independent

**Figure 2:**
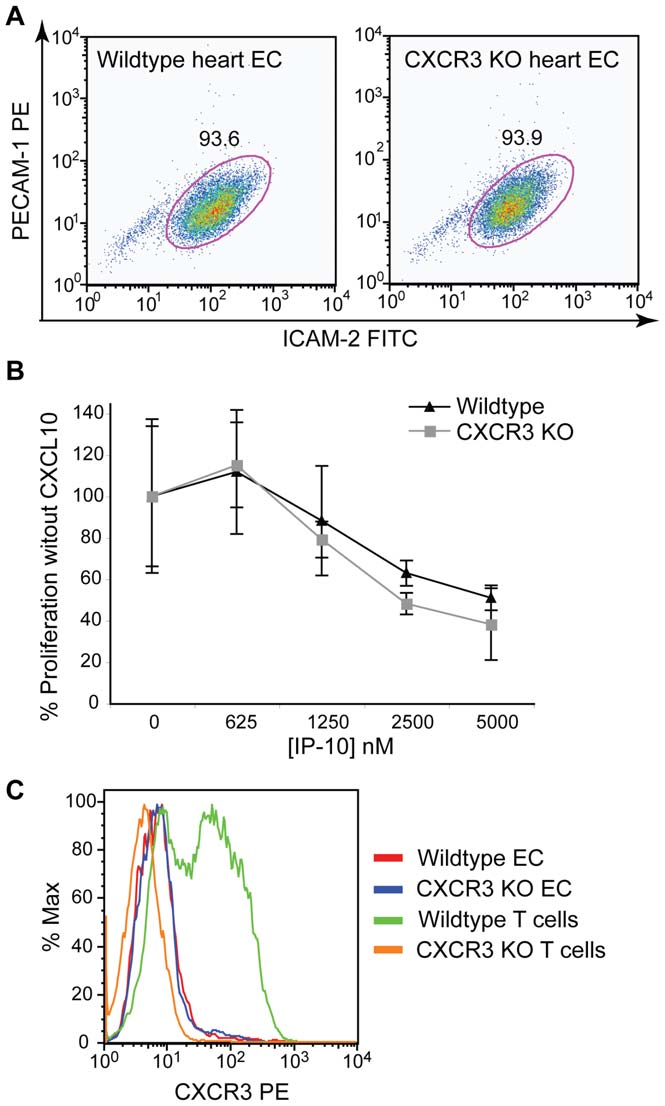
CXCL10 inhibits proliferation of CXCR3-deficient murine endothelial cells. A) Endothelial cells were isolated from hearts of wildtype and CXCR3-deficient (CXCR3 KO) mice and purity was determined by flow cytometry using antibodies against ICAM-2 and PECAM-1. Endothelial cells of at least 85% purity were used for all experiments. B) CXCL10's anti-proliferative effect was assessed as described for Figure 1. Representative data of at least three experiments are shown, each performed in triplicate, data are mean ± SD. C) CXCR3 expression of primary endothelial cells and activated T cells was assessed by flow cytometry. Wildtype heart endothelial cells did not express CXCR3, whereas activated wildtype T cells expressed high levels of CXCR3.

To directly test whether the anti-proliferative effect of CXCL10 requires CXCR3, we isolated endothelial cells from the hearts of wildtype and CXCR3-deficient (CXCR3 KO) C57Bl/6 mice. Endothelial cells were isolated using PECAM-1-coated microbeads, with a second selection utilizing ICAM-2-coated microbeads if needed. Endothelial cells were used for proliferation assays only if their purity was at least 85%, with purity levels often reaching over 90% (see Fig. 2A). CXCL10's anti-proliferative effect on bFGF-induced proliferation on primary heart endothelial cells was measured between passage 2 and 3. Both wildtype and CXCR3-KO endothelial cell proliferation were effectively inhibited by CXCL10 (Fig. 2B), clearly showing that CXCL10 can exert its anti-proliferative effect through a CXCR3-independent mechanism. We also analyzed the expression of CXCR3 on heart endothelial cells by flow cytometry and found no expression of CXCR3 on wildtype cells (Fig. 2C). In contrast, activated murine wildtype CD4+ Th1-type T cells expressed high levels of CXCR3.

### The alternative CXCR3-B splice variant does not exist in mice

**Figure 3:**
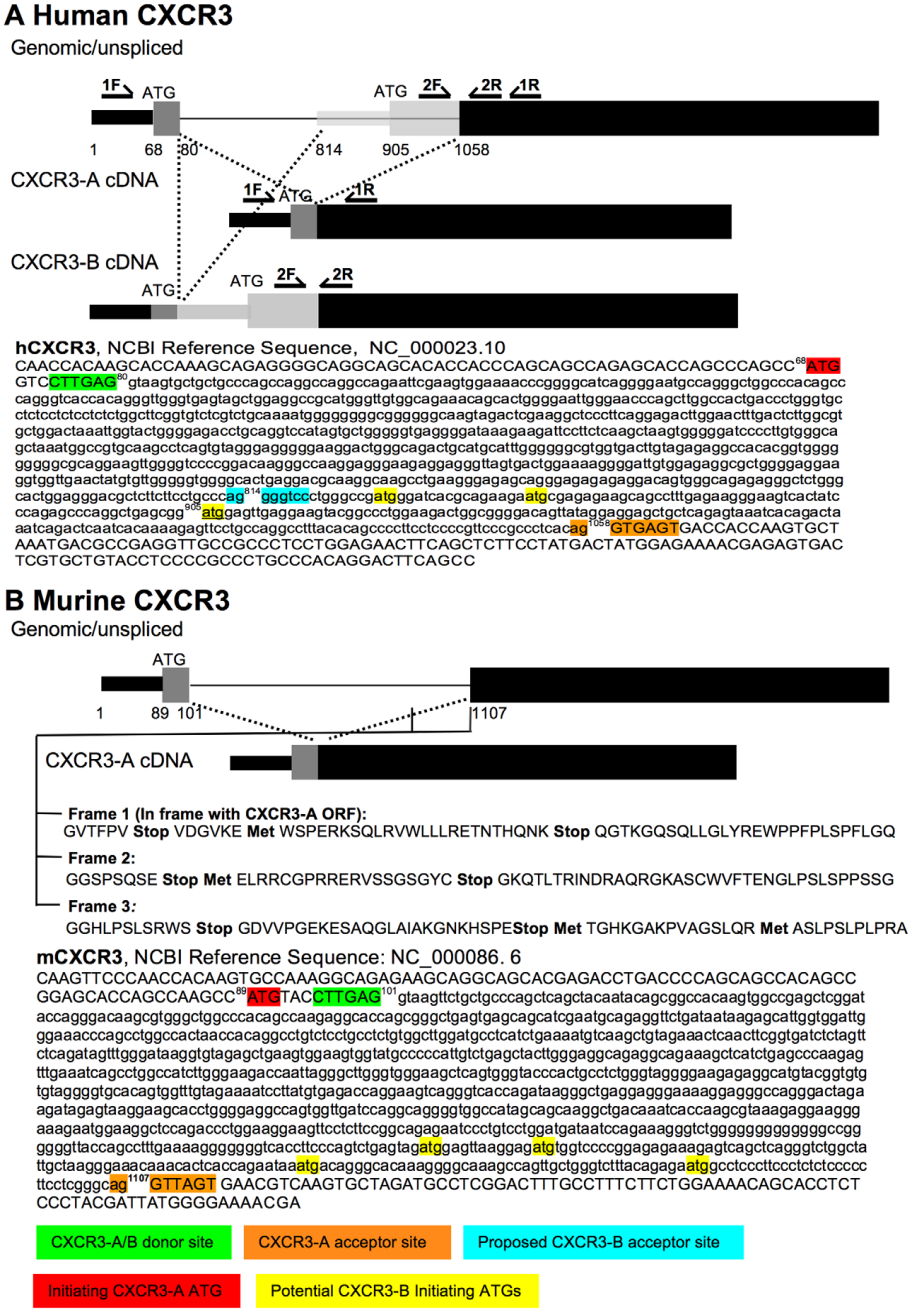
Alternative splice variant CXCR3-B does not exist in mice. A) human and B) murine CXCR3 schematic (top) and nucleotide sequence (bottom). The genomic/unspliced and cDNA are shown for CXCR3-A and B. Thick bars represent translated exons, thin bars untranslated exons, and thin lines introns. Regions common to both CXCR3-A and B are represented in black, regions that are specific for CXCR3-B in light grey. The 5′ region that is translated for CXCR3-A, but untranslated for CXCR3-A is shown in dark grey. CXCR3-A and CXCR3-B splice donor site is indicated in green, CXCR3-A splice acceptor site in orange, proposed human CXCR3-B splice acceptor site in cyan. Initiating ATG codon for CXCR3-A is indicated in red, and potential initiating ATG codons for CXCR3-B in yellow, with the proposed CXCR3-B ATG underlined. For human CXCR3, the primer pairs used for detecting CXCR3-A [1F (forward) and 1R (reverse)] and CXCR3-B (2F and 2R) are indicated in the schematic. For murine CXCR3-B, nucleotide sequence position 912–1112 was translated to assess whether CXCR3-B exists in mice using the Expasy Proteomics server (http://www.expasy.org/tools/dna.html). Frame 1 is in-frame with the remaining 3′ sequence, but a stop codon would terminate any possible murine CXCR3-B.

Since CXCL10 has angiostatic effects in mice as well as on human endothelial cells, we wanted to know whether alternative splicing could result in a CXCR3-B splice variant in mice. Both human and murine CXCR3 have a splice donor site six base pairs (bp) after the initiating ATG, with a 978 bp intron and 1006 bp intron, respectively, before the AG acceptor site (Fig. 3 A and B). This splice variant produces the originally identified CXCR3 cDNA, which is now sometimes referred to as CXCR3-A. The hCXCR3-B splice variant is produced by an alternative splice between the same donor site used by CXCR3-A (Fig. 3A, position 80), and a different acceptor site located 233 bp upstream of the AG acceptor site for CXCR3-A (position 814). Translation of this mRNA could be initiated from an alternative ATG start codon found within the intron of CXCR3-A (Fig. 3A, highlighted in yellow, position 905), 151 base pairs upstream of the known AG acceptor site used for intron splicing and in-frame with the remaining 3′ sequence of CXCR3-A. To determine whether a possible alternative splice site in the murine genome could result in a homologous CXCR3-B splice variant in mice, we analyzed the nucleotide sequence of the murine gene upstream of the known AG acceptor used for CXCR3-A splicing. For a homologous CXCR3-B splice variant to exist in mice, the translation would need to start with an initiating ATG in frame with the remaining CXCR3 sequence, without a stop codon terminating translation. In analogy with the human splice variant, we analyzed the 200 base pairs upstream from the murine acceptor site, and translated the open reading frame that would be in frame with the remaining 3′ translated sequence (Fig. 3B, frame 1). In this ORF, there is a potential initiating ATG at position 948, but a stop codon at position 1023 would prematurely terminate translation, and would therefore not result in a CXCR3-B splice variant with an alternative, longer N-terminal sequence. There are also a number of other ATG codons upstream of the 2^nd^ exon of CXCR3-A, but none of them are in frame with the remaining 3′ translated sequence (frame 2 and 3 in Figure 3B). Therefore, even if there was an alternative acceptor splice site in mice, it would not result in a translated CXCR3-B splice variant similar the one proposed in humans.

**Figure 4:**
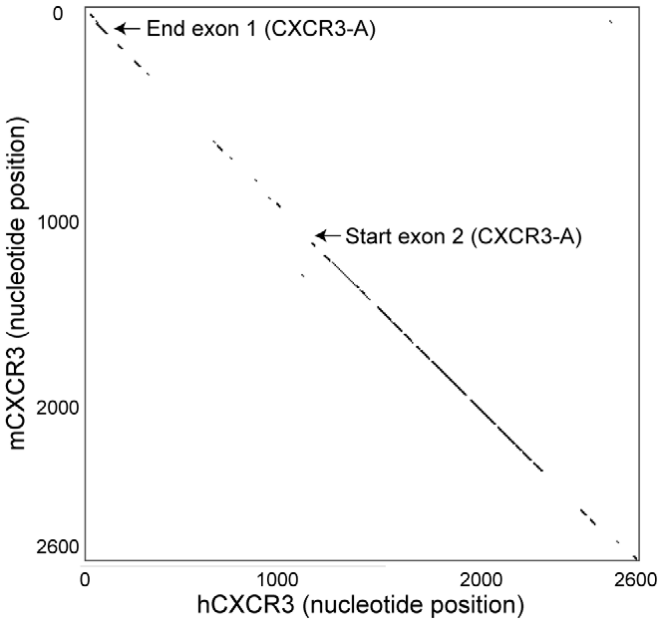
Homology of human and murine CXCR3 gene is confined to CXCR3-A coding regions. A dot matrix of the human CXCR3 and the murine CXCR3 gene was generated with the Dnadot software (http://arbl.cvmbs.colostate.edu/molkit/dnadot/), with a window size of 21 and a mismatch allowance of 3.

We next performed a sequence comparison between the human and murine CXCR3 genes to analyze which regions of the gene are conserved between the two species. If an orthologue of CXCR3-B were to exist in mice, a similar conservation would be expected in the CXCR3-B coding sequence as in the CXCR3-A coding sequences. We generated a dot matrix plot aligning the hCXCR3 and mCXCR3 gene on an X–Y plot (Fig. 4), using a window of 21 base pairs with a mismatch limit of 4. The conserved regions between human and murine CXCR3 clearly match to the 5′ region of exon 1, and the region of exon 2, starting at position 1066. In contrast, there is very low homology in the coding sequence of the proposed elongated hCXCR3-B N-terminus (starting at position 904), further suggesting that the murine gene does not share a homologous CXCR3-B splice variant. Finally, we performed a Blast search on the NCI EST (expressed sequence tags) database for murine genes with the 150 base pairs upstream of exon 2, which would be unique to a CXCR3-B splice variant, and found no matching cDNA clones. In contrast, using the cDNA sequence that crosses the first intron in the “traditional” CXCR3-A variant from exon 1 into exon 2, four cDNA clones were identified. Altogether, these three approaches strongly suggest that the murine CXCR3 gene does not contain a CXCR3-B splice variant similar to the one proposed in the human genome.

### HUVEC and HMVEC-L do not express CXCR3 yet are inhibited by CXCL10

**Figure 5:**
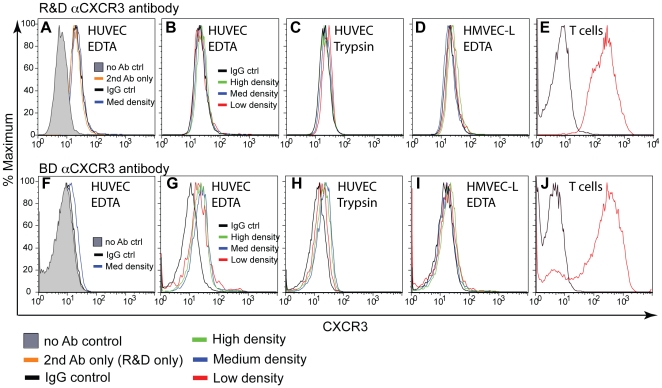
HUVEC and HMVEC-L have no detectable surface CXCR3 expression. CXCR3 surface levels were assessed by flow cytometry using anti-hCXCR3 monoclonal antibodies from R&D (A–E), or BD Bioscience (F–J), for HUVECS (A–C, F–H), HMVEC-L (D and I) and human peripheral blood T cells cultured in IL-2 (E and J). Endothelial cells were harvested at different densities to determine cell cycle dependency of CXCR3 expression (B–C, G–H), and were harvested either with EDTA (A, B, D, F, G, I) or trypsin (C and H).

There have been conflicting results about the expression of CXCR3 by endothelial cells [23]–[26]. We therefore investigated CXCR3 surface expression using flow cytometry on HUVEC and HMVEC-L, two human endothelial cell preparations that are sensitive to CXCL10's antiproliferative effects (Fig. 1). We utilized two different anti-CXCR3 antibodies: 1C6 from BD Bioscience, directly conjugated to APC, which is commonly used to detect CXCR3 expression on T cells; and an anti-CXCR3 antibody from R&D (clone 49801), which has been reported to detect CXCR3-B [22]. Since previous studies reported cell-cycle dependent expression of CXCR3 [23], [27], we analyzed CXCR3 expression on HUVEC and HMVEC-L harvested at high, intermediate or low cell density. Finally, we also compared the use of EDTA or Trypsin to release the endothelial cells from the tissue culture plate, as Trypsin could potentially cleave CXCR3 expressed on the cell surface. The R&D anti-CXCR3 antibody was not directly conjugated to any fluorophore, so cell binding was detected with a secondary antibody conjugated to PE. Staining HUVEC with the R&D antibody revealed a small shift in fluorescence compared to cells stained with no antibody; however, this shift was also present for cells stained with an isotype control antibody, or for cells stained only with the secondary antibody (Fig. 5A), clearly demonstrating that this small shift was not due to CXCR3 expression. We then compared the staining of HUVEC harvested at high, intermediate or low cell density, with either EDTA or Trypsin, and found no CXCR3 expression under any of these conditions (Fig. 5B–C). Similarly, staining of HMVEC-L, a different primary endothelial cell preparation, with the R&D anti-CXCR3 antibody, at different cell densities, utilizing either EDTA (Fig. 5D) or Trypsin (not shown), also revealed no CXCR3 expression. In contrast, staining of activated human T cells with the R&D anti-CXCR3 antibody demonstrated high levels of cell surface CXCR3 expression (Fig. 5E). The same sets of experiments were performed with the BD anti-CXCR3 antibody (Fig. 5F–J). A small shift in fluorescence was observed staining HUVEC with the BD antibody compared to the isotype control antibody (Fig. 5F). There was some variability in the isotype control background binding but in general this control antibody had very low background binding most likely because it was a directly conjugated antibody. We suspect that the slight shift of the BD antibody compared to the isotype control does not represent surface expression of CXCR3, and this slight shift was not dependent on cell cycle, EDTA or Trypsin treatment (Fig. 5G–H). For HMVEC-L, no shift in fluorescence was detected between the anti-CXCR3 antibody and the isotype control antibody using the BD antibody (Fig. 5I). In contrast, as a positive control, the BD monoclonal antibody detected high levels of CXCR3 surface expression on activated human peripheral blood T cells (Fig. 5J).

**Figure 6:**
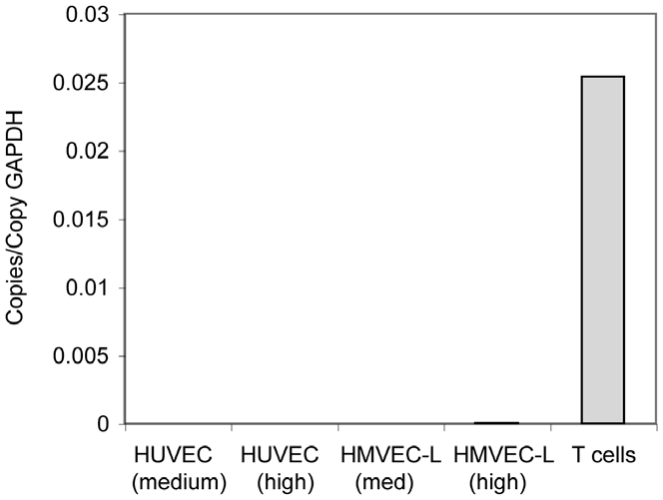
HUVEC and HMVEC-L have very low expression of CXCR3 mRNA. HUVEC and HMVEC-L were harvested at two different cell densities, and human peripheral blood T cells were harvested after 7 days in culture with IL-2. A primer pair in the common sequence in exon 2 of CXCR3 was used to detect both CXCR3-A and B splice variants using qPCR.

Expression of CXCR3 in HUVEC, HMVEC-L and T cells was further investigated at the mRNA level by quantitative PCR, using primers in the common region of exon 2 (forward primer position 1324–1345), which should detect both CXCR3-A and CXCR3-B. In agreement with the flow cytometry data, HUVEC and HMVEC-L, harvested at intermediate or high cell density, had very low signal for CXCR3 mRNA, whereas T cells had a very high signal for CXCR3 mRNA expression (Fig. 6). Together, these data show that the same endothelial cells that were inhibited by CXCL10 had no detectable CXCR3 mRNA or protein expression.

### CXCL10's anti-proliferative effect is not neutralized by two different anti-CXCR3 antibodies

**Figure 7:**
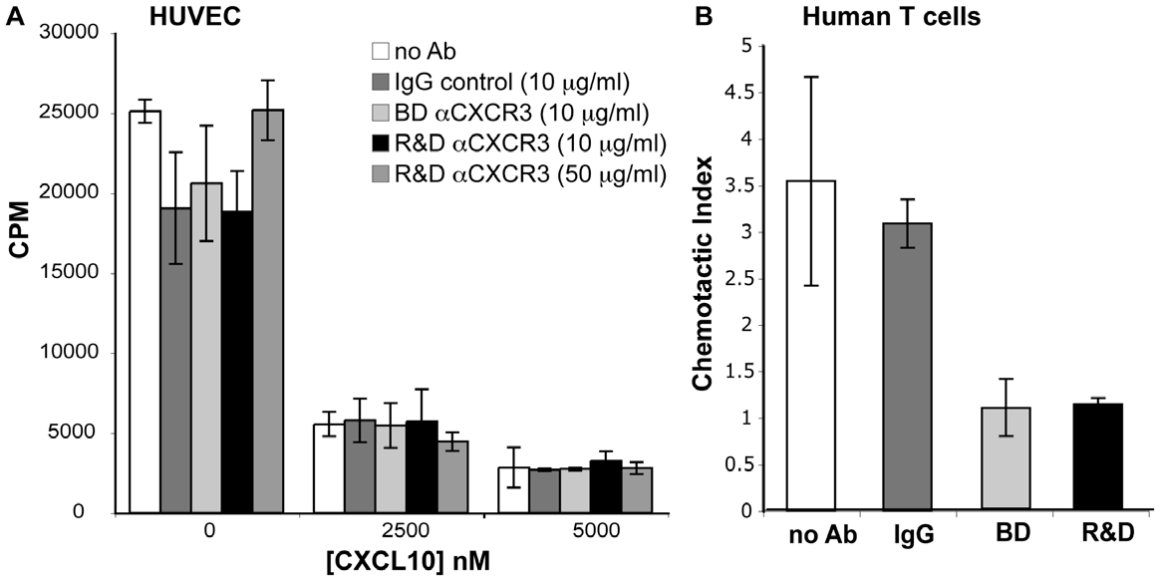
Neutralization of CXCR3 does not diminish CXCL10's anti-proliferative effect. A) HUVEC were plated in 96 well plates with bFGF. 24 hr after plating, new media with CXCL10 and anti-CXCR3 antibody from BD Bioscience (10 µg/ml) or R&D (10 or 50 µg/ml), or an isotype control IgG (10 µg/ml). ^3^H-thymidine incorporation was assessed for the final 18 hr of incubation. Representative data of two experiments are shown, each performed in triplicate, data are mean ± SD. B) BD Bioscience and R&D neutralizing anti-CXCR3 antibodies block the ability of CXCL10 to induce chemotaxis of human T cells. 100 ng/ml CXCL10 was placed in the bottom well of Neuropore chemotaxis chambers. Human T cells were pre-incubated with 10 µg/ml antibody, and were added on top of the Neuroprobe membrane and allowed to migrate for 2 hours. Representative data from two experiments are shown, each performed in duplicate, data are mean ± SD.

We next tested the ability of the two neutralizing anti-hCXCR3 antibodies to inhibit CXCL10's anti-proliferative effect on HUVEC. Endothelial cells were incubated with the R&D antibody (10 µg/ml or 50 µg/ml), the BD antibody (10 µg/ml) or an isotype control antibody (10 µg/ml) for the duration of the proliferation assay. The antibodies did not affect HUVEC proliferation in the absence of CXCL10, and had no effect on the ability of CXCL10 to inhibit HUVEC proliferation (Fig. 7A). The neutralizing activity of these antibodies was confirmed by their ability to inhibit chemotaxis of human T cells to CXCL10 (Fig. 7B). It is particularly important to note that the R&D anti-CXCR3 antibody has been reported by Lasagni et al. to neutralize CXCR3-B activity [22], yet in our hands, it did not inhibit the anti-proliferative effect of CXCL10 on HUVEC. These findings further support that CXCL10's anti-proliferative effect on human endothelial cells is not dependent on CXCR3.

### CXCL10's anti-proliferative effect correlates with glycoaminoglycan binding rather CXCR3 binding

**Figure 8:**
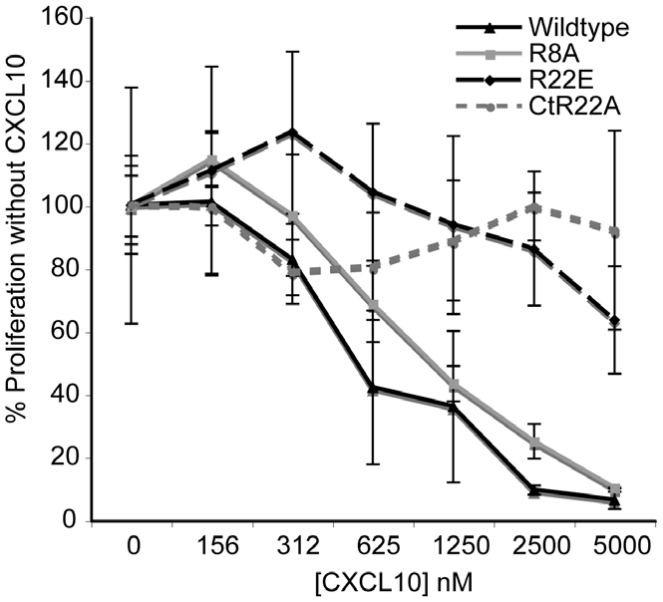
CXCL10's anti-proliferative effect correlates with GAG binding, but not CXCR3 binding. HUVEC were plated in 96-well plates with bFGF. 24 hr after seeding, new media with CXCL10 (wt or mutants) was added and replaced every 48 hr for a total of 3–4 days. Mutant R8A is devoid of CXCR3 signaling, but has normal heparin binding affinity. Mutant R22E has low heparin binding affinity and low CXCR3 signaling, and mutant CtR22A has minimal CXCR3 signaling and very low heparin binding affinity. ^3^H-thymidine incorporation was assessed for the final 18 hr of incubation. Representative data of at least three experiments are shown, each performed in triplicate, data are mean ± SD.

We next investigated the requirement of CXCR3 binding for CXCL10's anti-proliferative effect on HUVEC utilizing a series of CXCL10 mutants we previously described [28]. In particular, mutant R8A has greatly decreased CXCR3 binding affinity and does not induce chemotaxis or calcium-flux through CXCR3 *in vitro*, or recruitment of T cells *in vivo* [29], whereas it binds normally to glycosaminoglycans (GAGs). Mutant R8A was however fully able to inhibit HUVEC proliferation to the same extent as wildtype CXCL10 (Fig. 8), again suggesting that the anti-proliferative effect of CXCL10 on endothelial cells in this assay is not dependent on CXCR3 binding and signaling. In contrast, mutant R22E, a partial CXCR3 agonist, but with markedly reduced GAG binding affinity, had a minimal effect on HUVEC proliferation even at the highest concentration tested. Consistent with this, mutant CtR22A, which has four basic residues in the C-terminal helix mutated to acidic residues (K71E/R72Q/K74Q/R75E) as well as a mutation of R22A, and has very low GAG and CXCR3 binding, also did not inhibit endothelial cell proliferation. Taken together, these findings suggest that GAG binding rather than CXCR3 binding is important for CXCL10's anti-proliferative effect.

## Discussion

CXCL10 is one of the most highly induced cytokines during Th1-type inflammatory responses. CXCL10's activity on effector T lymphocytes and other leukocytes cells has been clearly demonstrated to be mediated by activation of its high affinity G protein-coupled receptor CXCR3. However, CXCL10 exerts effects on cells other than leukocytes, including endothelial cells. The mechanisms through which CXCL10 influences the activity of these cell types is not entirely clear. We therefore focused our study on the well-documented effect of CXCL10 on the proliferation of endothelial cells, employing a defined *in vitro* assay to study the anti-proliferative effect of CXCL10. Various mechanisms have been suggested to explain this anti-proliferative effect of CXCL10 on endothelial cells, including 1) signaling through CXCR3 on endothelial cells, 2) competition with growth factors for GAG binding, and 3) direct signaling of chemokines through GAGs.

The hypothesis that CXCL10's anti-proliferative effect on endothelial cells is mediated by CXCR3 received wider acceptance after the identification of a proposed alternative splice variant in humans called CXCR3-B, which was reported to specifically mediate these effects of CXCL10 [22]. However, there is still much debate whether CXCR3-B is indeed a functional receptor that mediates differential functions in contrast to the originally described CXCR3 (or CXCR3-A). Although a number of groups have reported human CXCR3-B on different cell types, they mostly rely on the same primer pairs described in the original publication by Lasagni et al. [22]. However, the CXCR3-B primer pair used in these studies does not span an intron and therefore will also detect and produce the same sequence for unspliced RNA. The forward primer used for detecting CXCR3-B is located after the alternative initiating ATG within the intron of CXCR3-A (position 1011–1029 within Pubmed gene reference NC_000023.10, see Fig. 3A) and the reverse primer is located within the exon (position 1089–1070), resulting in a PCR product of 79 bp. To our knowledge this fact has not been discussed in any prior publication. In contrast, the primers commonly used to detect CXCR3-A (including those used by by Lasagni et al.), span an intron; these primers are located before the donor splice site for the forward primer (position 40–58) and after the acceptor splice site (position 1128–1104). Importantly, this CXCR3-A primer pair should therefore give a product for spliced CXCR3-A *and* CXCR3-B, with a PCR product of 111 bp for spliced CXCR3-A and 355 bp for spliced CXCR3-B. However, in most publications using endothelial cells [22], tumor cells [30], [31] and epithelial cells [32], high levels have been reported with the CXCR3-B primers, but very low levels with the CXCR3-A primers, which has led to the conclusion that CXCR3-B is preferentially expressed on these cells. The question remains though why in these publications the CXCR3-A primers did not give a signal for CXCR3-B. We are furthermore concerned that when we performed a Blast search on the NCI EST (expressed sequence tags) database with the 5′ nucleotide sequence unique to human CXCR3-B no matching cDNA clones were identified in any of the published cDNA libraries. In contrast, using the 5′ cDNA sequence of human CXCR3-A that crosses the first intron in a similar Blast search of the NCI EST database, we identified 13 matching cDNA clones. In the original CXCR3-B publication, the authors describe that they identified a clone in a leukocyte cDNA library that showed complete homology with CXCR3-B, but this library does not seem to be available on-line anymore.

An important consideration regarding CXCR3-B is the efficiency with which the spliced human CXCR3-B mRNA would be translated. The first ATG codon within spliced CXCR3-B mRNA is the same initiating ATG of CXCR3-A (see Fig. 3A, highlighted in red). Since this is a strong Kozak consensus sequence [33], translation should start at this position. There are also two additional ATG codons before the proposed CXCR3-B translational start. All three of these distal ATG codons (see Fig. 3A, highlighted in yellow) have weaker Kozak sequences than the upstream CXCR3-A ATG (Fig. 3A, highlighted in red). It is therefore unlikely that translation would start at the proposed CXCR3-B ATG. In fact, the presence of upstream ATG codons is seen as a warning sign whether cDNA clones reflect a functional mRNA [33]. Translation of the putative human CXCR3-B splice mRNA is therefore expected to start at the CXCR3-A ATG codon, but this translation would get terminated after 22 codons. Theoretically, translation can get re-initiated, as eukaryotic ribosomes can resume scanning and reinitiating translation downstream of the stop codon [34]. The proposed CXCR3-B translation start site is the next ATG after the stop codon and could therefore result in re-initiation and translation of CXCR3-B. However, re-initiating is inefficient, particularly as the upstream open reading frame (ORF) lengthens past 13 codons, which in the case of CXCR3-B is 22 codons (66 base pairs). Studies have shown that very short upstream ORF (3–9 codons) reduced the yield to 30–35%, and 13–33 codons resulted in a further 3-fold lower efficiency [34], [35]. It is also important to note that the presence and abundance of mRNA or cDNA transcripts do not unequivocally establish the presence of functional mRNA and its efficient translation [33]. Indeed, many immature or partially spliced mRNAs have been found in cDNA libraries, in particular of G protein-coupled receptors [36], partly due to difficulties in cloning long transcripts. It has also been suggested that immature RNA, and especially slow or only partial removal of the 5′ intron is a way to regulate mammalian gene expression [33]. With this in mind, there is real concern whether CXCR3-B is expressed as a functional protein in non-transfected cells and whether it is expressed on the cell surface. The first publication by Lasagni et al. [22] produced two anti-CXCR3-B specific monoclonal antibodies, but these antibodies have not been made widely available, and have only been used in a few publications. While we deliberately chose not to focus our study on CXCR3-B, we do want to point out these uncertainties about the CXCR3-B splice variant and whether this variant explains the anti-proliferative and angiostatic functions of CXCL10 in human cells.

A further open question has been whether CXCR3-B exists in mice and other rodents. We have not found any published papers directly referring to CXCR3-B in mice, although many studies have reported angiostatic effects of CXCL10 in mice *in vivo* and *in vitro*. Analyzing the murine CXCR3 gene based on published sequences, we show here that a stop codon would terminate a potential in-frame CXCR3-B splice variant. In support of this conclusion, we found no sequences in the mouse EST database matching the putative mouse CXCR3-B specific region. Even if CXCR3-B mediates anti-proliferative effects in humans, there has to be a different explanation for CXCL10's angiostatic and anti-proliferative effects in mice.

Due to the complexity of potentially different pathways being involved in mediating CXCL10's *in vivo* angiostatic effect, we focused solely on the question on whether the *in vitro* anti-proliferative effect of CXCL10 on endothelial cells requires CXCR3. The clearest experiment to test the requirement of CXCR3 was to utilize CXCR3-deficient endothelial cells derived from mice in which the majority of exon 2 of the CXCR3 gene, which encodes almost the entire protein, has been deleted. We first showed that CXCL10 has the same anti-proliferative effect on primary murine heart endothelial cells as on HUVEC and HMVEC, the two main human endothelial cells used to investigate CXCL10's angiostatic effect. We next demonstrated that CXCL10 inhibits CXCR3-deficient endothelial cells as efficiently as wildtype endothelial cells, clearly pointing to a CXCR3-independent pathway for this effect. It is interesting to note though that various studies reported that the angiostatic effect of CXCL10 was reduced in CXCR3-deficient mice. In particular CXCR3-deficient mice displayed impaired post-ischemic neovascularization [20], and delayed and impaired wound healing [18], [19]. However, CXCL10's angiostatic effect in these models might not primarily be mediated by its direct anti-proliferative effects on endothelial cells, but mediated indirectly through its effects on other CXCR3-expressing cells. Indeed, Waeckel et al. demonstrated that in CXCR3-deficient mice, fewer T cells and monocytes/macrophages accumulated after ischemic injury and that transfer of wildtype bone marrow-derived mononuclear cells restored the impaired neovascularization in CXCR3-deficient mice, pointing to a leukocyte recruitment-dependent mechanism contributing to CXCL10's angiostatic effect *in vivo* [20].

We performed further experiments to test whether CXCL10's *in vitro* anti-proliferative effect on human endothelial cells is CXCR3 dependent. First, we analyzed CXCR3 expression by flow cytometry on the same endothelial cells that were inhibited by CXCL10. In our hands, using two different CXCR3 blocking antibodies, one of which was reported to detect CXCR3-B, we were not able to detect significant expression of CXCR3 on HUVEC or HMVEC-L. Quantitative PCR using primers specific to sequence in exon 2 common to CXCR3-A and CXCR3-B also revealed that HUVEC and HMVEC-L have very little, if any, CXCR3 mRNA expression. Furthermore, the two antibodies did not neutralize CXCL10's anti-proliferative effect on HUVEC. Both of these findings clearly point to a CXCR3-independent mechanism, in agreement with the fact that CXCL10 inhibited proliferation of CXCR3-deficient murine endothelial cells. Finally, we utilized a series of CXCL10 mutants we generated to map the CXCR3 and GAG binding sites of CXCL10 [28]. Mutant R8A, which is devoid of CXCR3 signaling but with retained GAG-binding, inhibited endothelial cell proliferation as well as wildtype CXCL10, strongly suggesting again that this effect is CXCR3 independent. A similar finding was also reported by Proost et al. with an N-terminally truncated CXCL10, which was devoid of CXCR3 signaling but retained an angiostatic effect in the rabbit cornea micropocket model [37]. In contrast, mutant R22E with markedly reduced GAG-binding affinity, had a minimal effect on HUVEC proliferation. This independent line of investigation suggests that the ability of CXCL10 to inhibit endothelial cell proliferation is more associated with its binding to glycosaminoglycans than its binding to CXCR3.

Indeed, one of the first explanations for CXCL10's and CXCL4 ‘s anti-proliferative effect on endothelial was that they interfered with the binding of growth factors to GAGs [12], [38]. Growth factors, in particular bFGF and VEGF, utilize GAG binding to aid binding to their high affinity receptors [39]. CXCL4 anti-proliferative effect has been clearly shown to include a component related to competition of heparin binding as well as heparin binding-independent components [40]–[43], which was however not related to CXCR3-B [43]. Similarly, CXCL10's strong heparin binding affinity could interfere with the binding of growth factors to GAGs, thereby not involving CXCR3.

A third possible mechanism suggested for CXCL10's anti-proliferative effect could be direct signaling through GAGs. Chemokine binding to GAGs has been demonstrated to cause direct signaling in a number of different studies. For example, CCL5 (RANTES) has been shown to activated the phosphotyrosine kinase (PTK)-dependent and p44/p42 mitogen-activated protein kinase (MAPK) signaling pathways through GAGs [44]. Similarly, the GAG component of the proteoglycan syndecan-4 has been identified as a signaling molecule for CXCL12 (SDF-1) [45].

While we suspect a component of CXCL10's antiproliferative effect on endothelial cells is the result of its affinity for GAGs and the resultant displacement of growth factors from the cell surface, we cannot exclude other mechanisms that may also contribute to its effects on endothelial cells. However, our data strongly suggest that CXCL10 can inhibit the proliferation of endothelial cells through a CXCR3-independent pathway, and that this should be considered when trying to understand the biology of CXCL10 and CXCR3.

## Materials and Methods

### Ethic Statement

Mice were bred and maintained and experiments were performed according to protocols approved by the Massachusetts General Hospital Subcommittee on Research Animal Care under the MGH institutional assurance number A3596-01.

### Materials, cells and mice

Human umbilical vein endothelial cells (HUVEC) and human lung microvascular endothelial cells (HMVEC-L) were obtained from Lonza, cultured with complete Lonza EGM-2 (HUVEC) or EGM-MV-2 (HMVEC-L) culture media and used between passage 3–6. C57Bl/6 wild-type mice were purchased from National Cancer Institute. Breeder pairs of CXCR3-deficient (CXCR3 KO) mice were a kind gift from Dr. G. Gerard (Children's Hospital, Harvard Medical School, Boston, MA). Recombinant CXCL10 (wildtype or mutant) was produced and purified as described before [28]. Antibodies were from BD, unless stated otherwise.

### Isolation of primary heart endothelial cells

Heart endothelial cells were isolated from wildtype or CXCR3 KO C57Bl/6 mice as previously described [46], [47], with slight modifications. Briefly, hearts were removed after intracardial perfusion with 30 ml of PBS, were cut very finely and digested for 45 minutes with 0.2% collagenase II (Roche) at 37°C with gentle shaking. The tissue was disrupted further by gentle tituration with a 14 g cannula and then filtered through a 70 µm cell strainer to obtain single cell suspensions. The cells were incubated with sheep anti-rat IgG Dynal beads (Dynal Corp), that were previously coated with anti-PECAM-1 antibody (BD Biosciences), for 10 minutes at room temperature with end-over rotation. Bead-bound cells were isolated with a magnetic separator, washed and plated on gelatin-coated flasks in complete DMEM, supplemented with 20% fetal calf serum, 100 µg/ml porcine heparin (Sigma) and 100 µg/ml endothelial mitogen (Biomedical Technologies). When the cells reached 70–80% confluency, the cells were detached with trypsin-EDTA and washed. Purity was assessed by flow cytometry using anti-murine PECAM-1-PE (BD Bioscience) and anti-murine ICAM-2-FITC. If purity was below 80%, cells were further purified by selection on anti-murine ICAM-2 antibody coated Dynal-beads. Endothelial cells over 85% purity were used for experiments between passage 2–3.

### Proliferation assay

Endothelial cells (HUVEC, HMVEC, primary murine heart endothelial cells) were plated into 96-well plates at densities of 1–4×10^3^/well in their respective media, with only bFGF as the growth factor at 5 ng/ml initially (R&D) [12], [38], and then at the concentration provided for in the Lonza EGM media kit without heparin supplementation in later experiments as this did not affect our results. One day after seeding the cells, the media was removed and replaced with media containing CXCL10. Two days later, the media was removed and replaced with fresh media containing CXCL10. The cells were cultured with CXCL10 for a total of 3–4 days, and [^3^H]-thymidine (NEN, 1 µCu/well) was added for the last 18 hr of incubation.

### Flow cytometry

Surface levels of CXCR3 protein on human endothelial cells or T cells was analyzed by flow cytometry, using two different antibodies. The first was an anti-hCXCR3 monoclonal antibody from R&D (clone 49801), used at 2 µg/stain, employing a mIgG_1_ isotype control (R&D), also at 2 µg/stain. The cells were incubated with the primary antibody for 25 min, washed and stained with a secondary goat- anti-mouse-PE labeled antibody (Caltag Laboratories, 2 µl/stain) for 20 min. The second antibody used to detect CXCR3 expression was a directly conjugated anti-hCXCR3 antibody from BD Bioscience (1C6, APC-conjugated, 10 µl/stain), employing a mIgG_1_-APC isotype control (BD Bioscience), staining the cells for 20 min. For CXCR3 expression experiments, endothelial cells were grown at three different densities, and harvested either with 0.25% trypsin or with 20 mM EDTA, between passage 3–6. Human T cells were purified with anti CD3-MACS beads (Milteny Biotech) and grown in complete RPMI, 10% FCS and hIL-2 (PeproTech) for 6–8 days.

Surface levels of CXCR3 protein on murine cells was analyzed using an anti-CXCR3-PE conjugated antibody from R&D (FAB1685P, 10 µl/stain). Murine endothelial cells were purified as described above. Murine T cells were isolated from the spleen and lymph nodes of wildtype and CXCR3-deficient C57Bl/6 mice with anti CD4 Dynal beads and grown under Th1 conditions with anti-CD3, anti-CD28, anti-IL-4, IL-12 and IL-2 for six days. Endothelial cells and T cells were stained with anti-CXCR3-PE antibody for 20 minutes, washed and flow cytometry was performed on a FACS Calibur and analyzed with Flow Jo software.

### Chemotaxis

Chemotaxis assays were performed as described [28]. Briefly, chemokine dilutions were added to the bottom well of a 96-well chemotaxis plate (NeuroProbe, 5 µm pore size). Human T cells were pre-incubated with anti-hCXCR3 antibody (R&D or BD, 10 µg/ml) or an isotype control (10 µg/ml) for 10 minutes and then added on top of the membrane (2.5×10^4^ cells) and allowed to migrate to CXCL10 (100 ng/ml) at 37°C for 2 h, after which cells in the bottom wells were counted under a microscope. The Chemotactic Index was calculated by dividing the number of cells that migrated in the presence of chemokine by the number of cells in the presence of buffer only.

### Quantitative PCR

Total RNA from HUVEC, HMVEC-L and human T cells prepared as above was isolated with an RNAeasy kit (Qiagen), converted to cDNA and analyzed by qPCR as described [48] using the MX4000 multiplex quantitative PCR system (Stratagene).
